# Editorial: What’s new in endocrinology? volume II

**DOI:** 10.3389/fendo.2025.1561375

**Published:** 2025-02-17

**Authors:** Jeff M. P. Holly, Marc R. Blackman, Sally Radovick, Hubert Vaudry

**Affiliations:** ^1^ School of Translational Health Science, Faculty of Medicine, University of Bristol, Bristol, United Kingdom; ^2^ Veterans Affairs Medical Center, Washington, DC, United States; ^3^ Department of Pediatrics, University of Arizona Health Sciences, Tucson, AZ, United States; ^4^ Department of Cell Imaging, University of Rouen-Normandy, Mont-Saint-Aignan, France

**Keywords:** endocrinology, growth hormone, IGF - I, obesity, diabetes mellitus type 2, Cushing’s syndrome, multiple endocrine neoplasia (MEN), melatonin

To mark a decade since Frontiers in Endocrinology was launched, in 2019 the Chief Editors commissioned a series of articles to reflect the dynamic activity in the field. Since then, our submissions have increased dramatically and we have now commissioned a second series to underscore the continuing evolution of endocrinology. These articles highlight recent breakthroughs or advances, new technologies, or challenges in the field of endocrinology, and exemplify some of the recent developments and new questions and challenges for the future. The publications cover many different areas of endocrinology, including issues related to some of the greatest health challenges facing today’s society, such as obesity, diabetes mellitus, reproduction, and ageing.

Ageing research is a relatively new and active field within endocrinology. This is partly due to shifting demographics, as increasing numbers of people survive longer into old age and the realisation that ageing is a regulated process and not merely a consequence of stochastic decay and accumulated damage. The endocrine system is central to this regulation and the growth hormone (GH)/insulin-like growth factor-I (IGF-I) axis appears to be critical for ageing, with evidence from many experimental models, both in non-humans and humans, indicating that any disruption of the axis results in markedly prolonged lifespan. Laron syndrome, caused by mutations in the GH receptor and consequent IGF-I deficiency, is one of the best-characterised human conditions in which the axis is disrupted, and Werner and Laron reviewed recent findings from studies of Laron Syndrome that may help inform the mechanisms by which GH/IGF-I affect ageing. A genomic analysis of patients with Laron syndrome identified thioredoxin-interacting protein (TXNIP) as one of the most upregulated genes; TXNIP plays a key role in several cellular processes involved in ageing, which include metabolism, apoptosis and senescence. In addition, a survey of microRNAs that are highly expressed in Laron syndrome patients identified upregulation of miR-132-3p, and a genome-wide analysis of its targets revealed that one of the genes under its inhibitory control was SIRT1, which is known to play a critical role in determining longevity. These studies of Laron syndrome have revealed new targets of the GH/IGF-I axis that may be important for the effects of the axis on ageing, opening up new avenues for further research.

The prevalence of obesity and type 2 diabetes mellitus (T2DM) have grown alarmingly across the world in the last few decades and these disorders have become major public health challenges. Both conditions are caused by hormonal dysregulation, and it has become increasingly clear that these result at least partly from dietary changes caused by transformations in our food environment. Anjom-Shoae et al. reviewed recent evidence examining the acute and long-term effects of high-protein diets and comparing the effects of diets high in either animal or plant protein. It is well established that high-protein diets can have beneficial effects on body weight and glycaemic control, at least in the short term, through their effects on satiety, gastric emptying times and gut hormone release. Diets high in either animal or plant protein exerted these beneficial effects on body weight and glycaemic control over shorter periods; in comparison, longer-term studies, greater than 12 months, indicated that diets high in plant protein had either neutral or beneficial effects, whereas diets high in animal protein exerted adverse effects. These latter observations are consistent with several prospective epidemiological studies of large populations, all of which have shown that diets high in animal protein are associated with an increased risk of obesity and T2DM. The reasons for the differential long-term effects of animal and plant proteins are not clear but may be due to differences in amino acid composition, glycaemic load and/or the greater insulinotropic effects of animal protein, with insulin then promoting fat deposition and inhibiting fat oxidation. Further studies are needed to understand the effects of different proteins on the gastrointestinal and other mechanisms involved in food intake and glycaemic control.

With the prevalence of T2DM continuing to increase, the marked differences in its presentation and progression pose a challenge to patient management. Groups working with the Risk Assessment and Progression of Diabetes (RHAPSODY) consortium have used an unsupervised, “bottom-up”, multimodal, multivariate strategy to analyse multi-omic data obtained from two European cohorts of patients with T2DM in order to better characterise the complexity of the molecular interactions underlying this heterogeneity among patients (Li et al.). By measuring 180 circulating lipids and 1195 proteins, they identified two subgroups of patients who differed in terms of insulin sensitivity and secretion, and glycaemic deterioration, and identified various groups of biomarkers, most notably those involved in immune processes, that were associated with these distinct subgroups of patients. These biomarkers will provide valuable targets for future studies examining causal pathways for disease progression.

Proinflammatory cytokines have been implicated in the pancreatic β-cell failure underlying both type 1 diabetes mellitus (T1DM) and T2DM. Understanding the molecular mechanisms involved in this process may lead to the discovery of biomarkers that can assist in monitoring disease progression and identifying new targets for therapeutic intervention. A novel cytokine-mediated effect on β-cell RNA turnover was reported by Ghiasi et al. Another highly international collaboration used human and rodent β-cell models to reveal a novel cytokine-mediated effect on β-cell RNA turnover. The latter investigators found that cytokines decreased the activity of nonsense-mediated RNA decay (NMD), which normally functions to eliminate prematurely terminated mRNAs, and thus could disrupt the balance between anti- and pro-apoptotic transcripts. These findings open up new avenues of research into the various components of the NMD machinery as potential biomarkers and/or therapeutic targets.

The regulation of reproductive function is intimately controlled by metabolic status. Recent advances in our understanding of the pathways involved were reviewed by Rodríguez-Vázquez et al., with a focus on the impact of metabolism on pubertal development and fertility. They described the pivotal role played by the hypothalamus in integrating central neuronal signals with hormonal signals, with peripheral metabolic hormones such as leptin, insulin, and ghrelin acting on Kiss1 and GnRH neurons to regulate pubertal development according to metabolic status. In addition, it has emerged that many cellular sensors also play a direct role in this integration: these include energy sensors such as AMPK, mTOR and SIRT1, along with several lipid sensing pathways, such as fatty acid receptors, fatty acid transport proteins, and nuclear receptors. Nuclear receptors such as peroxisome proliferator-activated receptors are differentially expressed in specific hypothalamic neurons, and when fatty acids are bound, these receptors are then translocated to the nucleus where they can activate or repress gene transcription. In addition, both bile acids (BAs), which are synthesised in the liver, and secondary BAs, which are formed after transformation by the gut microbiota, interact with the G protein-coupled receptor, TGR5, in the hypothalamus. A complex framework for the integral regulation of whole-body metabolism and reproductive function was described, which should enable further functional connectivity mapping of the hypothalamic neuronal and glial cells involved.

Cushing’s syndrome (CS) is a classic, but relatively rare, endocrine disease caused by excess cortisol secretion and is treated by surgical excision of the causative adrenal or pituitary lesion. Despite successful treatment a large proportion of patients in remission suffer from persistent fatigue, muscle weakness and sarcopenia. Recent evidence has increasingly implicated microRNAs (miRNAs) in skeletal muscle regulation and Seco-Cervera et al. conducted a pilot study to screen for circulating miRNAs that could be potential biomarkers of sarcopenia in a group of patients with CS who were in sustained biochemical remission. In the pilot study, confirmed in a second validation cohort, the authors found that miR-28-5p was upregulated in CS patients with sarcopenia compared to those without. Previous evidence indicates that miR-28-5p is a muscle-specific miRNA that contributes to myoblast proliferation, differentiation and regeneration, suggesting that there may be a functional link to persistent sarcopenia. These preliminary findings suggest that miR-28-5p may be a promising candidate to serve as a circulating biomarker indicating the risk of persistent sarcopenia in treated CS patients despite normalised cortisol levels, who may then benefit from adapted exercise programs to improve their quality of life and prevent future falls. More generally it may also indicate a fertile area for future investigation as a potential therapeutic target for age-related sarcopenia and various pathological, muscle wasting conditions.

Two manuscripts in this Research Topic described updated guidelines for the diagnosis and management of rickets and multiple endocrine neoplasia (MEN), two other classic endocrinopathies, particularly in light of advances in molecular medicine. Rickets is a heterogeneous group of skeletal diseases resulting from impaired mineralisation of growing bones due to nutritional or hereditary disturbances in calcium and phosphate homeostasis. On behalf of the Bone and Mineral Metabolism Group of the Italian Society of Pediatric Endocrinology and Diabetology, Baroncelli et al. presented practical guidelines for the diagnosis, treatment, and management of patients with rickets. Comprehensive recommendations that include algorithms were provided to ensure early differential diagnosis of nutritional and genetic forms of rickets based on biochemical findings and clinical and radiological examinations. Specific recommendations for multidisciplinary treatment programs for each distinct form of rickets were also provided, including for rare, hereditary forms of the disease.

Multiple endocrine neoplasia (MEN) is a group of diseases characterised by multiple tumours occurring in the endocrine tissues of an individual. Since the first form, MEN1, was reported 70 years ago three further forms, MEN2, MEN3 and MEN4 have subsequently been described. These syndromes were initially characterised by their clinical phenotype and their underlying genetic causes were subsequently discovered. Romanet et al. reviewed recent advances in the genetics, diagnosis and screening of the various forms of MEN. MENs are rare hereditary diseases that are transmitted in an autosomal dominant manner and not all cases fit into the classic MEN subgroups. Romanet et al. described guidelines for MEN diagnosis along with challenges and pitfalls of different sequencing strategies that can be applied. The authors (1) emphasised the strategy for management of index cases and presymptomatic genetic screening and counselling of relatives; (2) described advances in strategies to unravel gene-disease relationships including the value of using induced pluripotent stem cells to generate patient-derived spheroids, tumoroids and organoids to investigate the pathophysiology and potential drug responses; and (3) highlighted the need for national or regional large cohort studies to better understand these rare diseases.

Melatonin a classical hormone that is produced by the pineal gland, an outgrowth of the posterodorsal thalamus, is secreted predominantly at night, with a primary endocrine role in maintaining circadian rhythms throughout the body. Less well known is that the majority of melatonin is produced by extrapineal tissues, and Reiter et al. provided an update on the potential functions of this second source of melatonin. They estimated that less than 5% of the melatonin in the body is produced in the pineal gland, while the vast majority is produced in multiple tissues throughout the body and is not linked to dark/light cycles or secreted into the circulation, but acts locally, primarily in an autocrine, and possibly also in a paracrine manner. This extrapineal melatonin appears to be produced in the mitochondria, where it plays a role in free radical scavenging, re-dox homeostasis and anti-inflammation, and may therefore be important in maintaining general health and counteracting pathology.

The final article in this Research Topic does not relate to advances in endocrine science or practice but describes an emerging challenge in the way endocrinology is reported. At Frontiers in Endocrinology, we have witnessed an exponential rise in the submission of manuscripts that are derivative, or primarily redundant and otherwise of low quality. This phenomenon has been exacerbated by manuscripts that do not report novel findings or new data, but instead report analyses of vast amounts of data that can now be found in large publicly-available databases. Additionally, there has been a considerable growth in redundant bibliometric reviews and fraudulent manuscripts, many of which are produced by “paper mills”. Such manuscripts have been observed in the majority of medical specialities and are of limited value to our or other readers. In a commentary, we document how this has affected Frontiers in Endocrinology, and we outline some of the measures that we have taken to reduce their frequency and prevent them from overwhelming the literature (Tobias et al.).

Technological advances continue to provide endocrinologists with ever more powerful tools that reveal new insights and opportunities for novel treatments, even for the best-characterised endocrine conditions. The challenges faced by endocrinologists also continue to evolve as populations increasingly age and modern lifestyles increase the burden of metabolic disease. This Research Topic reflects a variety of these challenges and the dynamic nature of modern endocrinology.

